# Can We Add Auricular Composite Graft to Our Rhinoplasty Armamentarium?

**Published:** 2013-01

**Authors:** Ali Manafi, Amir Eslami Shahr Babaki, Golnoush Mehrabani, Abtine Shahlaee, Amir Manafi

**Affiliations:** 1 Department of Plastic Surgery, Tehran University of Medical Sciences, Tehran, Iran;; 2 Sport Medicine Research Center, Tehran University of Medical Sciences, Tehran, Iran;; 3 Stem Cell Research and Transgenic Technology Research Center, Student Research Committee, Shiraz University of Medical Sciences, Shiraz, Iran; 4 Medical Student Scientific Research Center, Tehran University of Medical Sciences, Tehran, Iran;; 5 Medical Student, Shahid Beheshti University, Tehran, Iran

**Keywords:** Auricular composite, Graft, Armamentarium, Alar rim, Reconstruction, Rhinoplasty

## Abstract

**BACKGROUND:**

The ala of the nose, with its particular texture and characteristics, poses both aesthetically and functionally intriguing challenges and is rather problematic regarding choices for reconstructive methods. Both flaps and grafts have been used to restore natural structure of nasal ala. The present study summarizes a ten-year experience of reconstructive surgery using small composite grafts from non-cartilage bearing tissues, and large composite grafts, containing cartilaginous tissue, with a mean follow-up of 4 years and 8 months.

**METHODS:**

Cumulatively 56 patients were reported. Some of them required surgery due to previous cosmetic rhinoplasty. In 47 of the cases, a small graft from the non-cartilage bearing junction of ear lobule to helical rim sufficed. Nine patients had rather large defects for which grafts were harvested from the helical root. Donor sites were primarily closed and grafts were implanted in place in a single, rapid session.

**RESULTS:**

All small grafts had excellent take. Of 9 large grafts, 5 had excellent take, three had acceptable, and one, in a male smoker, failed to take. During follow-up, no gross deformity or poor scar was detected in either donor or recipient site.

**CONCLUSIONS:**

We have demonstrated that using both large and small auricular composite grafts has favorable long term results for reconstruction of alar rim deformities. However, use of small grafts seems more beneficial and applicability of large grafts requires further studies.

## INTRODUCTION

Aesthetic rhinoplasty is considered as one of the most common surgeries in Iran. Cosmetic nose surgery or nasal beautification is still the most common surgical operation of the authors. Secondary rhinoplasty comprises about 40% of these operations. Many of these secondary cases were shown to have radical alar base resection which may compromise external nasal valve function.^1^

The alar rims are fragile and complex structures. Their unique size, height, thickness and symmetry form the natural nasal appearance and function. The specialized skin which supports and supplies these complex structures provides competence of the external nasal valves and patency of the inlets to the nasal airways.^1^^-^^3^ The most common causes of alar rim distortion include trauma, congenital malformations, anatomical variations such as alar cartilage malposition,4 surgical interventions and cosmetic rhinoplasty. All these factors might alter the symmetry and contour of alar rims and prevent their ability to perform their role as external valve stabilizers.^4^ Skin replacement^5^ and cartilage or bone grafts^6^^-^^9^ have been used successfully for reconstructive operations in many instances. However, as the alar rims provide both skin cover and external valvular support, preservation of both functions is required. Therefore, autologous grafts that simultaneously replace both the cutaneous and cartilage deficiencies are often required for replacing the alar rim. Composite skin/cartilage grafts and skin/dense subcutaneous tissue/skin grafts harvested from the ear provide an ideal material for such reconstructive surgeries. Patients with abnormality of alar rims or excessive alar base resection are challenging cases to reconstruct.

We present a decade-long experience with composite grafts, consisting of skin/ dense subcutaneous tissue/ skin from non-cartilage bearing pinea between the helical rim and lobule of the auricle, to restore the normal appearance and function of the alar rim.

## MATERIALS AND METHODS

**Fig. 1:**
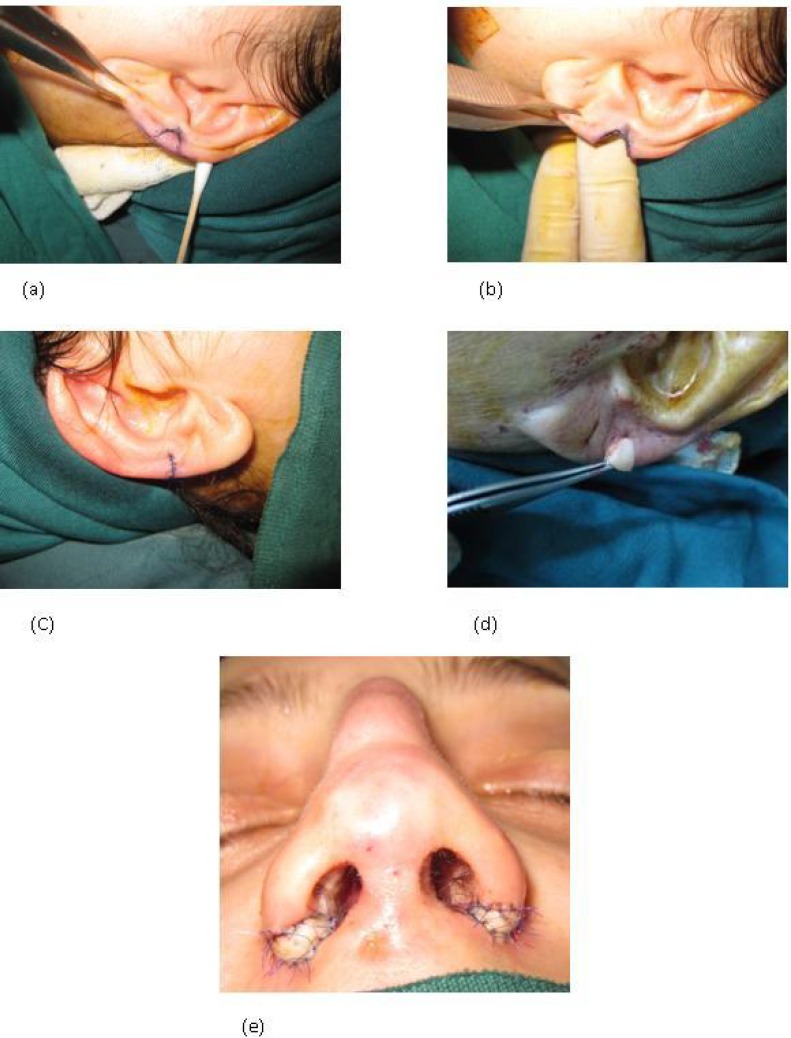
A suitable graft size is marked at the junction of helix and lobule. (b) The graft was harvested, (c) the donor site was closed primarily, and (d) the composite graft (e) was placed at the incised alar rim defect resulting in normal appearing nostrils

This prospective case-series study was pertinent to 56 patients with alar rim malformation, who presented between 2001 and 2011. The major causes of alar rim malformation in the study population were iatrogenic causes and trauma, that is, small and stenotic nostrils due to extensive alar base resection during previous rhinoplasty. Mean length of follow-up was 4 years and 8 months, with a maximum of ten years in some cases. All reconstructive procedures were performed using open approach. In 47 patients who had undergone previous rhinoplasty and needed small grafts, a wedge shape composite graft was harvested from the junction of the ear lobule to helix, as shown in Figure 1. The graft was used in conjunction with secondary rhinoplasty techniques for reconstruction of the whole nasal deformity.

As demonstrated, the site of previous incision in alar base was incised with a Number 15 blade to the required extent (Figure 1-a). The donor site was primarily closed. The graft was placed in position and sutured to the recipient site (Figure 1-e) In order to achieve both aesthetic and functional improvement of alar structure and shape. In the remaining 9 patients, a large graft was required and the composite grafts were harvested from the helical root. The composite grafts were implanted in either the alar rim defect or in the site of previous extensive alar rim resection, as well as the missing part of the alae.

## RESULTS

**Fig. 2:**
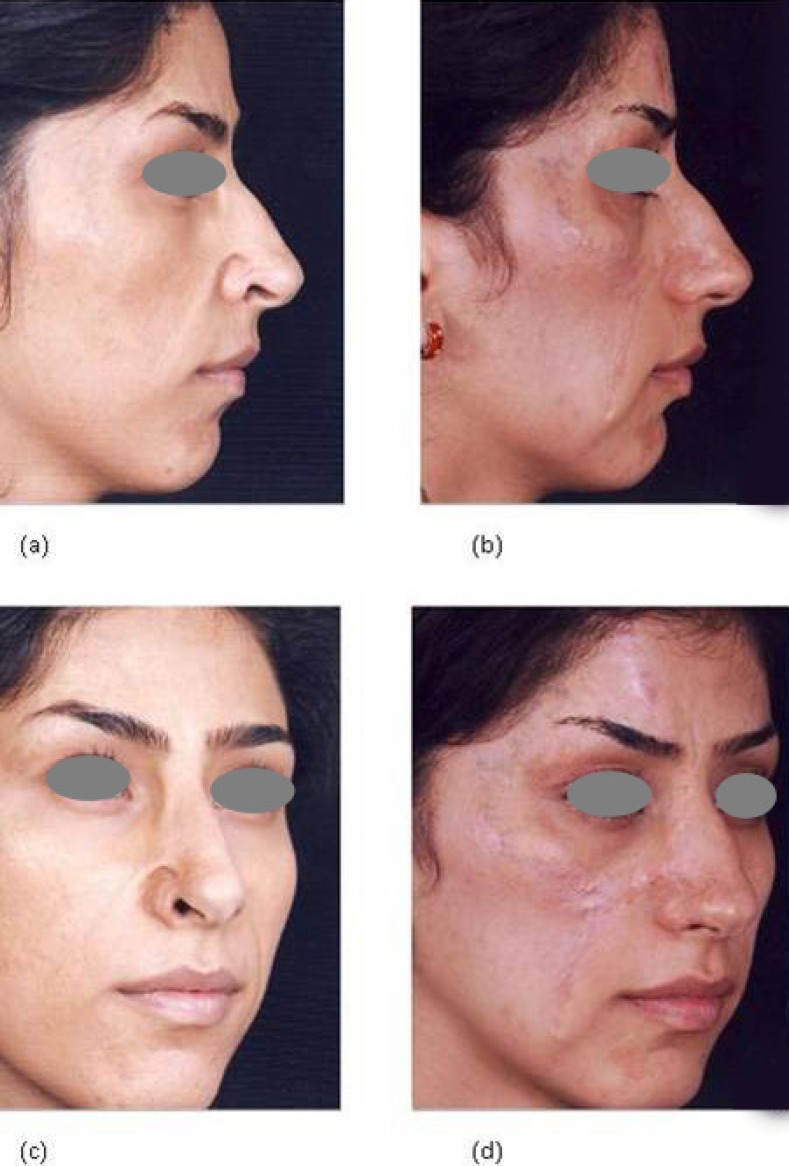
A patient with facial burn scars, with a defect at the right alar margin, on profile view, (a) before and (b) after reconstruction with composite graft and z-plasties. Images (c) and (d) are three-quarters view of the same patient demonstrating acceptable take and appearance

**Fig. 3:**
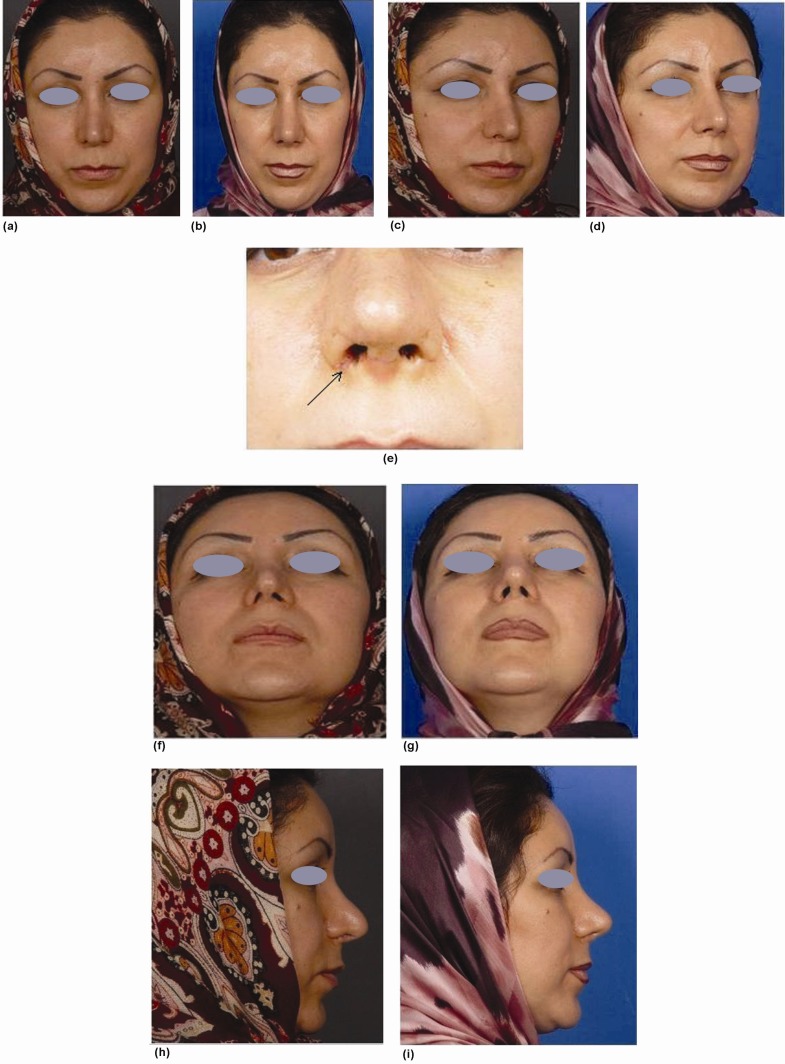
A lady who complained of asymmetric nostrils and other deformities following rhinoplasty. Images (a) and (b) show her on frontal view, before and after tertiary rhinoplasty and surgical correction with small composite grafts on the right alar rim, respectively. Figures (c) and (d) show the same patient, on three-quarters view. Figure (e) shows a close-up view of the same patient 10 days after surgery. The site of graft is indicated by an arrow. Figures (f) and (g) show basal view (h) and (i) show profile view

**Fig. 4:**
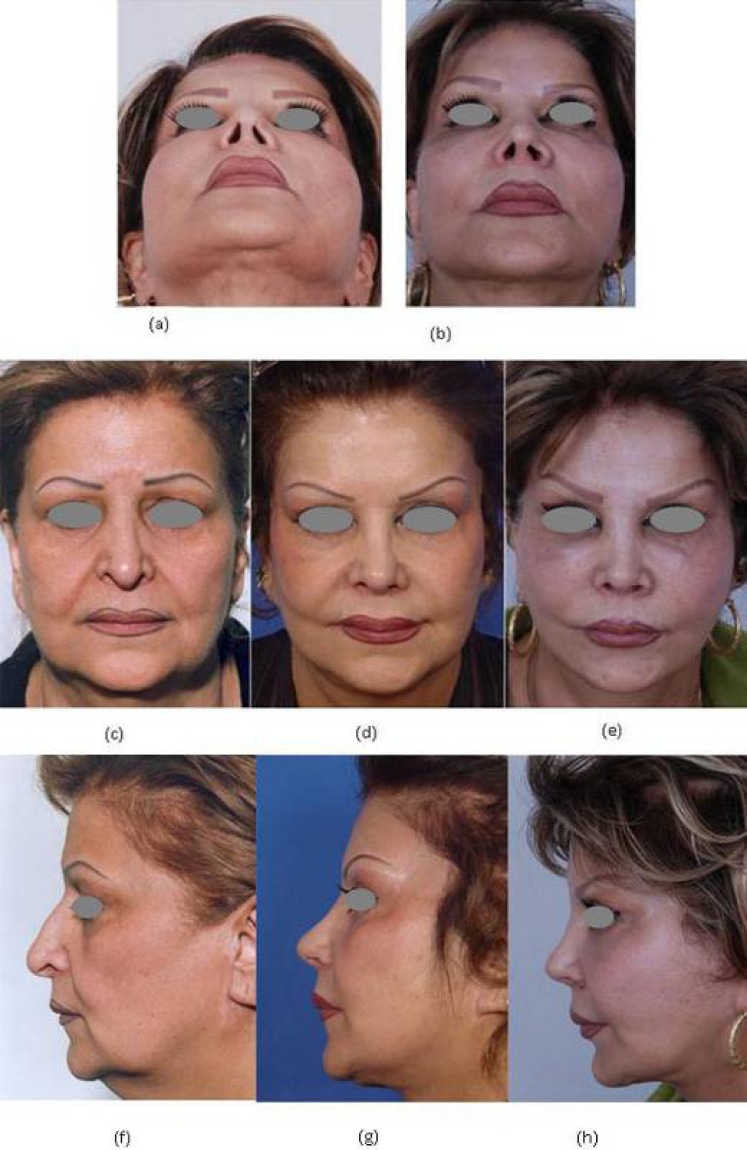
A lady presenting with asymmetry in nostrils in addition to dissatisfaction with previous rhinoplasty and face-lift, (a) before, and (b) after secondary rhinoplasty, reconstruction with a small composite graft, face-lift and lipoplasty, on frontal view. Additional images show frontal views of her, when presenting for the first time (c), after secondary rhinoplasty and face-lift (d), and three months after lipoplasty and implementing a composite graft on the right ala (e). Images (f), (g) and (h) show the same individual on profile view

**Fig. 5:**
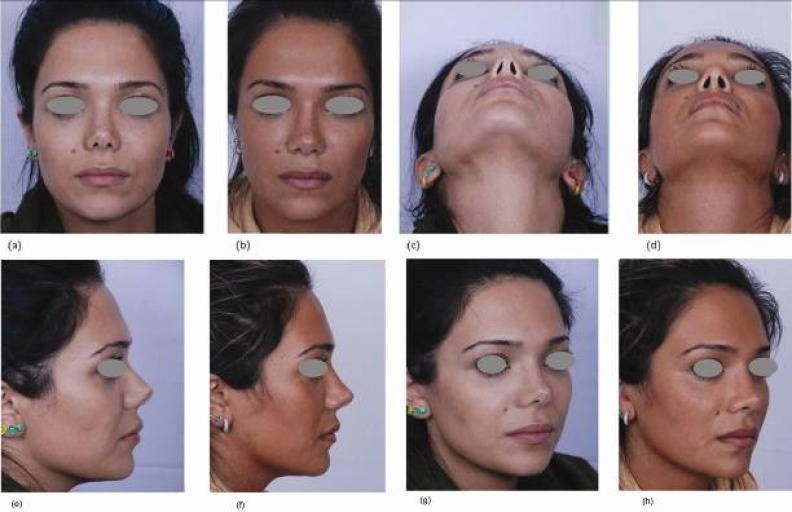
A young lady who had undergone two previous rhinoplasties, and the surgical and unnatural appearance of nasal tip and nostrils was her main complaint. Images (a) and (b) show her after tertiary rhinoplasty and composite grafting, on both sides, on frontal view. Figures (c) and (d) show her on basal view (e) and (f) show profile view (g) and (h) show three-quarter view

Fifty six patients with a mean age of 22 years (range between 17 and 62 years) made up our study population. All small grafts had excellent take and satisfying appearance for patients without obvious deformity of donor site. Five of the large grafts had excellent take, 3 of them had acceptable take and one failed to take. The pre-operative and post-operation pictures of four patients were shown in Figures 2-5.

## DISCUSSION

The ala is an important component of nasal anatomy, both aesthetically and functionally. Prior to attempting to re-establish the anatomy and functions of lost skin and skeletal structures, these defects should be carefully assessed. This approach may be beneficial for not only attaining desired results in surgery, but also for preventing fibrosis and contracture. Composite grafts are complexes of full thickness skin and surrounding periosteum and cartilage or skin/dense subcutaneous tissue/skin.^10^^-^^12^ Composite graft from either the helical rim or the root has been recommended for reconstruction of alar rim defects.^13^ Sangavi presented a case report of a 16 year-old girl with isolated congenital alar defect who underwent reconstruction with auricular composite graft. Composite auricular graft resulted in an excellent nasal contour correction without healing abnormality or any obvious deformity in the donor site.^12^ Coban and his colleague used the root of helix as the composite graft donor site for reconstruction of post-burn alar rim defect.^14^ Constantian used auricular composite graft reconstruction in 100 secondary and tertiary rhinoplasty patients.^15^ In his series, 99% of the grafts survived in their entirety and only two patients had partial unilateral graft loss. Moreover, Klinger and colleagues reported reconstruction of a full-thickness alar wound in a 20 year-old man using an auricular conchal composite graft16 which resulted in a complete repair of the defect with excellent wound healing as well as good functional and aesthetic results. However, the basis of treatment in these cases is resection of scar tissue or deformed ala, then grafting a piece of tissue with a 3-dimensional shape similar to normal anatomy to the alar area defect. These procedures are complicated and time-consuming, require a great deal of expertise, and it is not always feasible to harvest tissue with such characteristics. Moreover, there are challenges such as failure to take and healing abnormalities at the donor as well as recipient site. In the present series of 56 patients, we evaluated long term results of composite graft take in patients undergoing alar rim reconstruction. Our results demonstrated that composite graft has favorable results in alar rim reconstruction. In the present study, we utilized two different sets of grafts. For small defects or for individuals with congenital or acquired nostril stricture, a small wedge-shaped part of (non-cartilage bearing) helico-lobular junction, consisting of dense subcutaneous tissue in the middle and skin on both sides was used. This composite graft had excellent take in all 47 patients who needed small grafts. Due to limited manipulation, injury of donor site was very limited and no gross deformity or scar was observed in any of these patients. The other type of graft was harvested from the helix root. It was similar to the more traditional composite graftsin that it contained cartilaginous tissue.^6^ These grafts were needed to reconstruct alar rims in 9 patients who had relatively larger defects. We observed that in 5 cases the grafts had excellent take. In 3 individuals the grafts had acceptable take but in one patient, who was a male smoker, the graft failed to take. It has been suggested by other authors that auricular composite graft used for reconstruction of the alar rim should not be larger than 1.5 to 2 centimeters in diameter^17^ to ensure reliable revascularization. This supports our results which showed that in 9 patients who needed large composite grafts, only 5 patients had excellent graft take Where as all 47 patients with small grafts had excellent graft take. The main advantage of composite graft is that it can be performed in a single, fast surgical procedure with excellent contour correction.^12^ The main disadvantage of composite graft is that its use for large defects (larger than 2 centimeters) has not been recommended, and other therapeutic modalities such as nasolabial or forehead flap can be performed for these defects.^18^^,^^19^ Moreover, the final color of composite graft may not be very satisfying.^12^ In conclusion our results demonstrated that using auricular non-cartilage bearing composite grafts has favorable long term results in reconstruction of alar rim deformities. Although this holds true for both large and small grafts, it seems that there is the possibility of further improving the results of large grafts.

## CONFLICT OF INTEREST

The authors declare no conflict of interest.

## Footenote

Please cite this paper as:

Manafi A, Eslami Shahr Babaki A, Mehrabani G, Shahlaee A, Manafi A. Can We Add Auricular Composite Graft to Our Rhinoplasty Armamentarium? World J Plast Surg 2013;2(1): 33-40.
